# Prediction of anisotropic property of activated metal inert gas welding by employing different supervised machine learning models^☆^^☆☆^

**DOI:** 10.1016/j.mex.2025.103514

**Published:** 2025-07-26

**Authors:** Ruturaj U. Kakade, Nitin Khedkar, Amol Dalavi

**Affiliations:** a Department of Mechanical Engineering, Symbiosis Institute of Technology, Symbiosis International (Deemed University), Pune 412115, India; b MIT World Peace University, Pune 411038, India

**Keywords:** IG welding, linear regression (LR), Random forest regression (RFR), Support vector regression (SVR), Tensile strength prediction

## Abstract

Activated Metal Inert Gas (A-MIG) welding of EN10028 (SA516Gr70) steel was investigated using varying current, voltage, and gas flow rate to assess their influence on tensile strength (TS). A total of 100 welded samples were prepared and tested per ASTM standards. Material characterization was performed on samples with the highest and lowest TS to evaluate the correlation between microstructure and strength. Machine learning models Linear Regression, Random Forest Regression, and Support Vector Regression (SVR) were applied to predict TS based on welding parameters.

• The SVR model achieved the best predictive performance, with an R² of 0.8750 and a model accuracy of 96.73 %.

• The results confirm the potential of SVR for accurately forecasting TS in A-MIG welded EN10028, facilitating process optimization in pressure applications

## Graphical abstract

**Figure:**
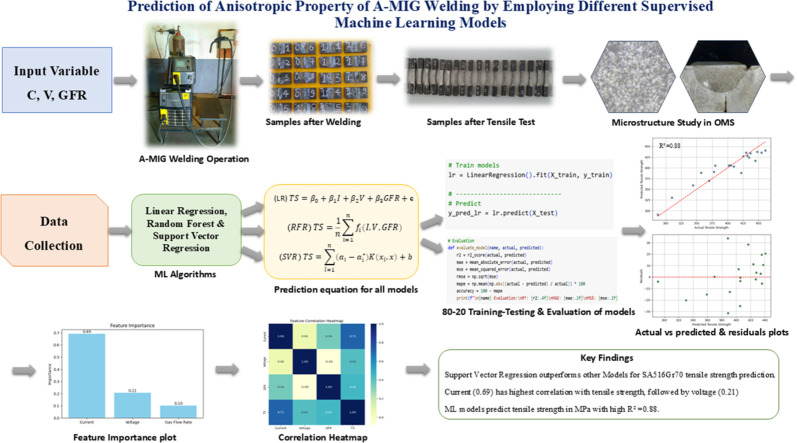

## Specifications table

**Table:** 

**Subject area**	Engineering
**More specific subject area**	Mechanical Engineering
**Name of your method**	Linear Regression, Random Forest Regression, Support Vector Regression
**Name and reference of original method**	Linear Regression (Montgomery, 2012), Random Forest Regression (Breiman, 2001), Support Vector Regression (Drucker et al., 1997)
**Resource availability**	The experimental dataset and Google Colab scripts used in this study are available from the corresponding author upon reasonable request.

## Background

This study focuses on enhancing tensile strength (TS) prediction in Activated Metal Inert Gas (A-MIG) welding, a widely used process in industrial applications, especially for boiler and pressure vessel fabrication due to its high productivity and ease of automation. Activated MIG welding improves penetration and mechanical properties, yet optimizing welding parameters and achieving desired TS remains challenging, particularly for EN10028 (SA516Gr70) material. An experimental design methodology was employed, varying current, voltage, and gas flow rate to prepare samples. Post-welding, specimens were tested for tensile strength following ASTM standards, and material characterization was also performed with the highest and lowest tensile strength to understand the correlation between weld structure and tensile strength. The study investigates machine learning models such as linear regression, random forest regression and support vector regression to precisely predict TS. Model selection is emphasized as critical for distinguishing between regression, classification, or clustering tasks. Data was split into training and testing sets for performance evaluation using metrics like R², MAE, MSE, RMSE, MAPE, and Accuracy. Additionally, actual vs. predicted TS plots, residual plots, feature importance, and correlation heatmaps were analyzed. This methodology offers a robust approach for TS prediction, supporting process optimization and quality control in critical welding applications.

Table 1 below is summary of studies for machine learning to predict tensile strength in welding and additive manufacturing across various materials. It highlights material used, ML models employed and best-performing results in each.

**Table 1:** Literature Review summery.

Study Focus	Material/System	ML Techniques Used	Best Model/Result	Reference No.
Predict tensile strength of welded AA2014-T6	AA2014-T6 Aluminium Alloy	SVR, Model comparison	SVR (R² = 0.89)	[1]
Neural network prediction of TS	Aluminium (chemical comp.)	ANN	RMSE = 15.383	[2]
Mechanical property prediction in metal AM	Additive Manufacturing (AM)	Not specified	–	[3]
Predict tensile strength in Monel 400 weldments	Monel 400	Random Tree, RF, C4.5, GB	Gradient Boosting (R² = 0.99)	[4]
Neural network to predict tensile strength	Austenitic Stainless Steel (MIG)	ANN (various training algorithms)	–	[5]
Plasma-MIG hybrid welding prediction	Plasma-MIG welded joints	Regression, ANN	–	[6]
Neural network to analyze MIG welding parameters	MIG welding (general)	ANN	UTS predicted	[7]
Predict TS of titanium alloys	Titanium Alloys	KNN and others	ML linked alloying to strength	[8]
Neurosymbolic prediction of UTS	Additive Manufactured Specimens	ANN vs Neurosymbolic	Neurosymbolic (R² = 0.9871)	[9]
ANN for rusted steel plate TS	Rusted Steel Plates	ANN + FEM inputs	–	[10]
Predict TS during FSW of aluminium alloys	Aluminium (FSW)	Adaboost classifier	Accuracy = 81.6 %	[11]
Ensemble ML for FSW UTS prediction	Friction Stir Welding	SVM, GPR	Enhanced accuracy	[12]
Boosting model for dissimilar FSW joints	AA6082-AA5083 (FSW)	Ensemble Boosting	R² = 0.985, RMSE = 5.292	[13]
GPR for maraging steel YS prediction	Ferrium PH48S	Gaussian Process	Fast with uncertainty quant.	[14]
Six ML models on FSW UTS & hardness	FSW joints	Deep Learning ANN best	–	[15]
Classifier-based tensile prediction in AM	CM247LC (AM)	ML Classifiers	Max TS = 997.81 MPa, Accuracy = 77.5–92.5 %	[16]

## Method details

**Fig. 1:**
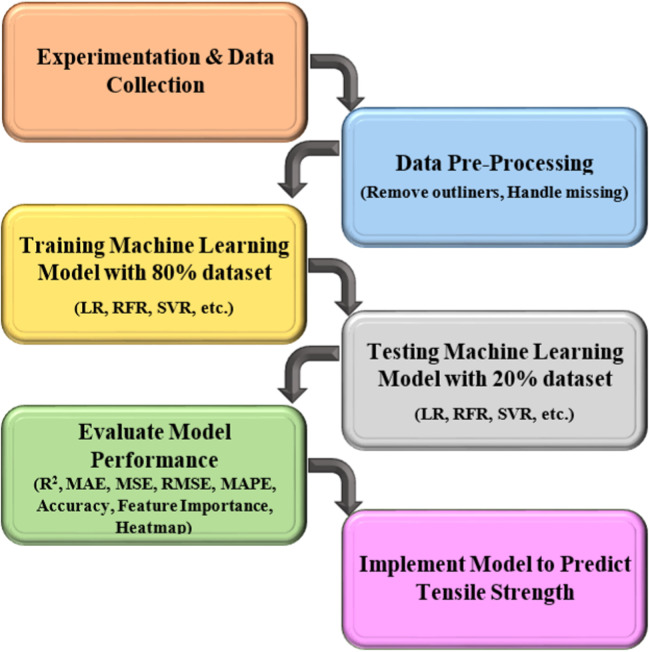
Flowchart indicating the research methodology followed in this study.

The methodology outlines the systematic approach adopted for the study. It includes the sequence of steps, experimental procedures, and analytical techniques employed throughout the research. The overall workflow of the method is illustrated in Fig. 1.

### Experimental setup

**Fig. 2:**
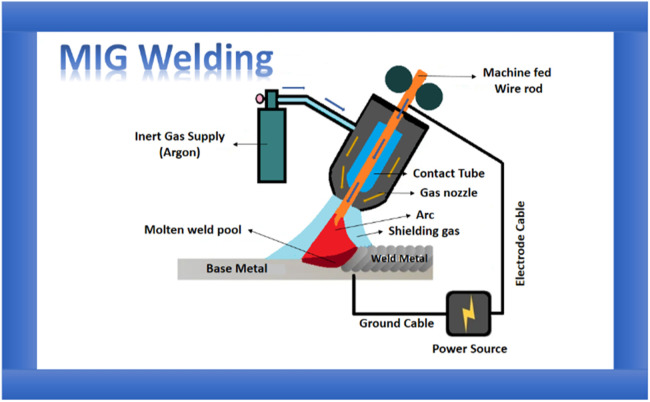
Schematic diagram of the Metal Inert Gas (MIG) welding setup.

**Fig. 3:**
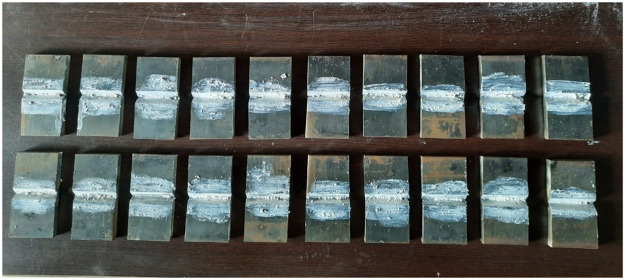
Specimen samples prepared for welding.

**Fig. 4:**
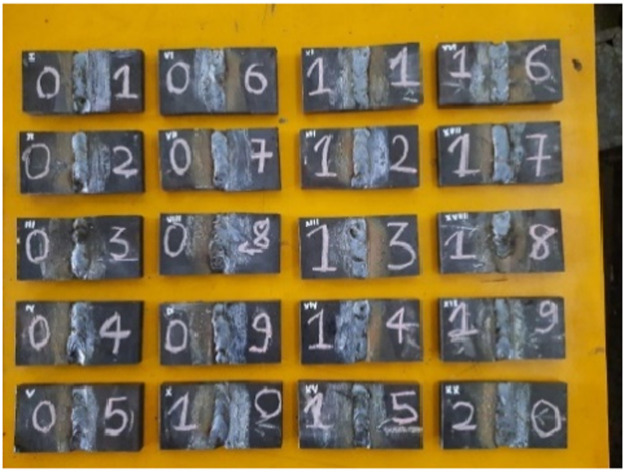
Specimen samples after welding.

The base metal employed in the current paper is EN10028 (SA516Gr70), a carbon steel plate with a dimension of 100×50×10 mm. Consumables selected during an investigation are ER70S6 with a diameter of 1.2 mm using the welding process operation as per the design of experiments and in the specified range of current, voltage, and gas flow rate. The range is selected based on a literature survey. Schematic diagram of the MIG welding setup and setup specifications are shown in Fig. 2 and Table 2, respectively. A metal inert gas welding was used with the welding working parameters specified in Table 3. Table 4, Table 5 summaries the chemical and mechanical specifications of the base parent and filler wire, respectively. The samples are prepared with a V notch of 45 degrees and spot welded to maintain the root gap. To improve the weld performance, ethanol was applied to the surfaces of welding samples, followed by painting with a 0.2–0.3 mm thick coating of activated flux material. Fig. 3, Fig. 4 show the preparation of sample specimens and the samples after the weld process.

**Table 2:** ARISTO MIG 400 Setup Specification.

SR. No.	Parameter	ARISTO MIG 400
1	Primary voltage (1 ph)	380 V 50/60Hz
2	Primary voltage (3 ph)	380–460 V 50/60Hz
3	Max. output	400A
4	Power factor at max current	0.95
5	Efficiency at max current	89.5 %
6	Fuse slow	20A
7	Energy sever mode	40W
8	Current range	16–400A
9	Dimensions	445×610×250mm
10	Electrode wire diameter	1.2–2.5mm

**Table 3:** Range of working parameter.

Parameters	Range
Filler material	ER70S6 (1.2 mm)
Welding current (A)	145–195
Welding voltage (V)	17–23
Gas flow rate (L/min)	09–13
Wire Feed Rate (m/min)	3.0–4.5
Material Thickness	10 mm
Weld Samples Parameter	
Length	100 mm
Width	50 mm
Root gap (mm)	1 mm
Welding position	1 G flat position

**Table 4:** Chemical composition of SA516Gr70 and ER70S6.

Element	C	Mn	P	S	Si	cu	Ni	Cr	Mo	V
SA516Gr70	0.27	1.10	0.025	0.015	0.55	0.30	0.30	0.25	0.08	–
ER70S6	0.09	1.50	0.025	0.035	0.80	0.50	0.15	0.15	0.15	0.03

**Table 5:** Mechanical Properties of EN10028 (SA516Gr70) and ER70S6.

Property	SA516Gr70	ER70S6
Density (*1000kg/m2)	7.86	7.85
Elastic modulus (MPa)	217	210
Tensile strength (MPa)	510–650	483
Yield strength (MPa)	355	≥ 400
Elongation ( %)	20	20

The welding variables selected in this investigation are Current, Voltage, and Gas flow rate. The shielding gas composition is 100 % argon. Single passes were required to fill the V notch. All samples were welded under a 1 G flat position.

### Tensile tests

Tensile strength is the peak stress a material can endure when subjected to a pulling force before failure occurs. This property is critical for determining a material's mechanical performance in structural applications. The value is derived from a tensile test, where the maximum load experienced by the specimen before fracture is divided by its original cross-sectional area [20]. In this study, tensile test experimentation is done to analyze the quality of the welded joints and compute the maximum tensile force required to break the weld. The tensile testing was carried out on a specimen in the I-section and perpendicular to the weld axis. The formula for Tensile Strength calculations is given below.

(1) $$\text{Tensile}\;\text{Strength}\;\left(\text{UTS}\right)=\frac{F_{\text{max}}}{A_{0}}$$

Where $F_{\text{max}}$​ Maximum force applied before fracture (N), $A_{0}$ Original cross-sectional area of the specimen (mm²)

**Fig. 5:**
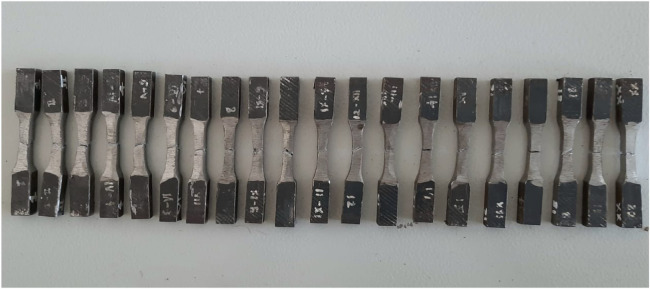
Tensile test specimens after tensile testing.

The samples were prepared for tensile test as per the ASTM A370 standard. The tensile testing was performed on each sample using a Computerized Universal testing machine capable of a maximum load up to 100T to measure higher tensile force and select the best sample. Fig. 5 shows the fractured samples after the tensile test. The sample dataset, consisting of welding runs with input current, arc voltage, shielding gas flow rate, and their corresponding tensile strength (TS) measurements, is provided in Table 6.

**Table 6:** Sample Dataset of tensile test results.

Experiment No.	Welding Current (A)	Welding Voltage (V)	GFR (L/min)	T.S (MPa)
1	170	20	11	410
2	150	18	12	339
3	190	22	10	431
4	150	22	12	425
5	170	20	11	405
6	190	22	12	432
7	190	18	10	396
8	170	20	12	405
9	170	20	11	402
10	170	22	11	448
11	167	19	11	373
12	172	18	11	376
13	195	17	12	405
14	171	18	10	415
15	173	23	10	445
16	193	17	11	401
17	154	18	9	300
18	150	18	12	345
19	170	22	10	425
20	146	18	9	282

### Microstructure testing

**Fig. 6:**
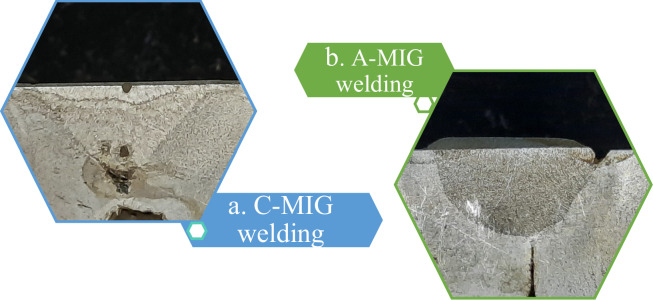
Weld bead profile of the joint showing bead geometry and surface characteristics.

**Fig. 7:**
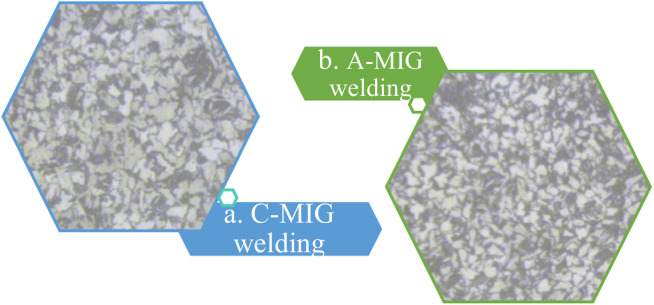
Optical microstructure of weld bead showing grain morphology.

Microstructure testing was performed on the samples that exhibited the highest and lowest tensile force in tensile testing. In order to assess the impact of Activated Metal Inert Gas (A-MIG) welding on Conventional Metal Inert Gas (C-MIG) welding, an extensive metallographic investigation was performed on cross-sectional weld specimens that demonstrated elevated tensile strength resulting from both methodologies. The samples were meticulously prepared utilizing standardized metallographic protocols and subsequently analyzed through optical microscopy. The microstructural analysis indicated that A-MIG welds exhibited a smooth, semi-elliptical bead configuration with complete penetration and a distinctly defined fusion boundary. Conversely, C-MIG welds presented a broader bead with less depth of penetration. The A-MIG specimens' heat-affected zone (HAZ) appeared more symmetrical and uniform, suggesting enhanced thermal regulation attributable to the activated flux. These findings substantiate the augmented arc stability and penetration efficacy of A-MIG welding. The weld bead profile of the MIG welded joint for a. C-MIG and b. A-MIG is shown in Fig. 6. Microstructural examination of the weld bead was performed to analyze the grain structure and phase distribution. The optical micrograph of the weld bead for a. C-MIG and b. A-MIG welding is presented in Fig. 7.

Microstructural examination of the weld reveals a uniform distribution of fine grains in A-MIG welding, whereas coarse grains are observed in C-MIG. Refined grains suggest a high cooling rate facilitated by enhanced arc concentration due to the activated flux. This grain refinement contributes positively to enhancing mechanical properties like tensile strength and toughness.

### Machine learning models

All machine learning algorithms, involving Linear Regression, Random Forest Regression, and Support Vector Regression, were implemented, trained, and tested with 80:20 ratio, where 80 samples were used for training the models (learn relationships between input parameters and tensile strength), while 20 samples were reserved for testing to evaluate the model's predictive performance on unseen data using Python in the Google Colaboratory (Colab) environment. Using Google Colab provided a cloud-based platform ensuring consistent execution and reproducibility of the results.

#### Linear regression (LR)

A basic and easily understood statistical method for simulating the linear connection between a dependent variable and one or more independent variables is linear regression. Carl Friedrich Gauss and Adrien-Marie Legendre, who created the least squares approach in the early 19th century, are credited with giving rise to linear regression. In MIG welding, LR can predict tensile strength (TS) based on input variables including Arc current, Arc voltage, and shielding gas flow rate. Despite its simplicity, it is a strong baseline model and provides insights into feature importance via model coefficients. However, LR assumes linearity, homoscedasticity, and independence of residuals, which can limit its effectiveness in modelling complex, nonlinear welding behavior [17].

The prediction equation for linear regression can be expressed as:

(2) $$\text{TS}=\beta_{0}+\beta_{1}I+\beta_{2}V+\beta_{3}\text{GFR}+\epsilon$$

Where TS is the predicted tensile strength, $\beta_{0}$is the intercept, $\beta_{1},\;\beta_{2},\;\beta_{3}$ are regression coefficients, I, V, GFR are the input variables, ϵ is the random error term.

**Fig. 8:**
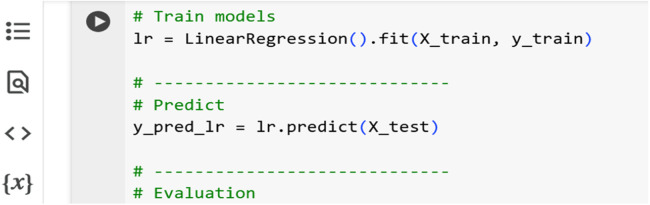
Python code for linear regression model.

The LR algorithm was applied to the original training data to establish a baseline relationship between input welding parameters and tensile strength. Predictions were made on the test set, and evaluation metrics were computed. Fig. 8 shows a screenshot of the Python code for a linear regression model.

#### Random forest regression (RFR)

Random Forest Regression (RFR), presented by Leo Breiman in 2001, is a combined model learning approach that builds multiple tree classifiers on bootstrapped data segments and takes the mean of their predictions to enhance accuracy and reduce overfitting. It establishes random variation by selecting a random sample of variables at each split, promoting model diversity and generalization. RFR effectively captures nonlinear relationships and complex parameter interactions without assuming any underlying data distribution. This makes it ideal for modelling experimental datasets, such as predicting tensile strength from welding parameters. Moreover, it offers built-in feature importance metrics, aiding in identifying key variables influencing output. RFR handles noise and multicollinearity well, ensuring stable and interpretable predictions even in high-dimensional spaces. Its robustness, flexibility, and interpretability make it a preferred choice for regression tasks in engineering and material science domains. RFR handles missing values and outliers effectively, though it can be computationally intensive for large datasets and offers less interpretability than linear models [18].

The prediction equation for Random Forest can be expressed as:

(3) $$\text{TS}=\frac{1}{n}\sum_{\overset{˙}{i}=1}^{n}f_{i}\left(I,V,\text{GFR}\right)$$

Where $f_{i}$ denotes the prediction from the ith decision tree, n is the number of trees in the forest.

**Fig. 9:**
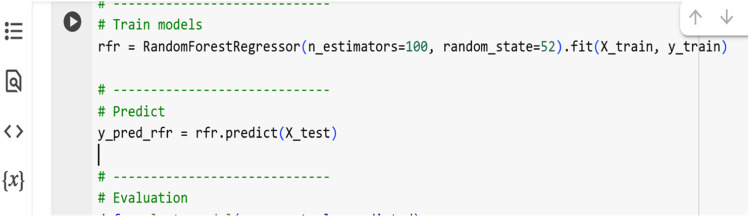
Python code for Random Forest Regression model.

The unscaled training data implemented a Random Forest Regressor comprising 100 estimators. This ensemble-based method improves prediction robustness by reducing variance. The model also provided feature importance insights. Fig. 9 shows a screenshot of the Python code for Random Forest Regression.

#### Support vector regression (SVR)

Support Vector Regression (SVR), developed by Vladimir Vapnik in the 1990s, represents an enhanced extension of support vector machine techniques, tailored explicitly for regression tasks. The core idea behind SVR is to determine a function that can approximate the target values within a certain tolerance level (denoted as ϵ), all while keeping the model as simple as possible. It uses kernel functions such as linear, polynomial, or radial basis functions to map input data into higher-dimensional feature spaces. This allows SVR to capture both straightforward and highly nonlinear patterns effectively. This characteristic makes SVR especially suitable for working with small to moderately sized datasets that may have many features. In welding, where the link between process variables (like current, voltage, and gas flow rate) and mechanical properties (such as tensile strength) is often complex and nonlinear, SVR provides reliable prediction accuracy and strong generalization ability. Its formulation, which emphasizes margin-based learning and the kernel trick, offers robustness in modelling intricate relationships with limited data [19].

The prediction equation for SVR model can be expressed as:

(4) $$\text{TS}=\sum_{\overset{˙}{l}=1}^{n}\left(\alpha_{\overset{˙}{i}}-\alpha_{\overset{˙}{i}}^{*}\right)K\left(x_{i},x\right)+b$$

Where $\alpha_{\overset{˙}{i}},{\;\alpha }_{\overset{˙}{i}}^{*}$ are Lagrange multipliers, $K(x_{i},x)\;$is the kernel function, b is the bias term, $x_{i}=\left[I,\;V,\;\text{GFR}\right]$ are training samples, n is the number of support vectors.

**Fig. 10:**
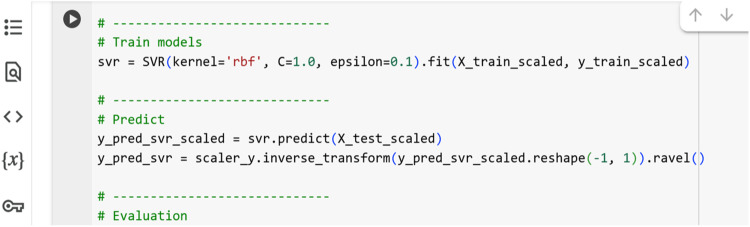
Python code for Support Vector Regression model.

However, the performance of SVR heavily depends on the choice of hyperparameters, namely the penalty parameter CC, the epsilon ϵ\epsilon in the loss function, and kernel-specific parameters which must be carefully selected through tuning. In this study, SVR was implemented using the RBF kernel, with input features standardized before training. After generating predictions, the results were converted back to the original scale of tensile strength using inverse transformation. Fig. 10 provides a snapshot of the Python code for implementing the SVR model.

## Method validation

### Measure metric

**Fig. 11:**
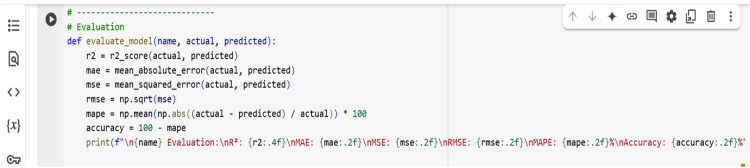
Python code for model evaluation.

Different regressions, such as linear regression, were implemented in the current investigation to evaluate forecast performance using 80 % training and 20 % testing. After this model performance evaluation is checked with a coefficient of determination value (R²), Mean Absolute Error (MAE), Mean Squared Error (MSE), Root Mean Squared Error (RMSE), Mean Absolute Percentage Error (MAPE), and Prediction accuracy to evaluate how well each model performs. The formulae to calculate the same are given below. A study of actual vs predicted and residuals vs predicted tensile strength for different ML models, feature importance, and correlation heatmap plots is generated in Google Colab. A screenshot of the Python code for model evaluation is shown in Fig. 11.

(5) $$R^{2}=1-\frac{\sum_{i=1}^{n}\left(y_{i}-{\hat{y}}_{i}\right)}{\sum_{i=1}^{n}{\left(y_{i}-\bar{y}\right)}^{2}}$$

(6) $$\text{MAE}=\frac{1}{n}\sum_{i=1}^{n}\left(y_{i}-{\hat{y}}_{i}\right)$$

(7) $$\text{MSE}=\frac{1}{N}\sum_{\overset{˙}{I}=1}^{N}{\left(y_{i}-\bar{y}\right)}^{2}$$

(8) $$\text{RMSE}=\sqrt{\left(\frac{1}{n}\sum_{i=1}^{n}\left(y_{i}-{\hat{y}}_{i}\right)\right)}$$

(9) $$\text{MAPE}=\frac{100}{n}\sum_{i=1}^{n}\left|\frac{y_{i}-{\hat{y}}_{i}}{y_{i}}\right|$$

(10) ACCURACY=100%−MAPE%

Where $y_{i}$ = Actual value, ${\hat{y}}_{i}$​ = Predicted value, $\bar{y}$​ = Mean of actual values, n = Number of observations, *N* = Number of data points

### Evaluation of machine learning algorithms

Table 7 compares the performance of 3 regression models to predict tensile strength. Linear regression performs worst, with a lower R2 value of 0.7438 and higher error values (MAE of 20.04, RMSE of 23.85, and MAPE of 5.20 %), leading to slightly reduced accuracy of 94.80 %. Random Forest Regression achieved a good level of accuracy with an R2 value of 0.8255, suggesting that it describes 83 % of predictions mostly close to the real values. It has a mean absolute error of 15.41, a root mean squared error of 19.69, and a mean absolute percentage error of 3.84 %, resulting in an overall accuracy of 96.16 %. Support Vector Regression outperformed the other two, demonstrating the best predictive capability with an R2 value of 0.8750, which means it explains nearly 88 % of the predictions mostly close to the real values. It also had the lowest error rates (MAE of 12.93, RMSE of 16.66, and MAPE of only 3.27 %), resulting in the highest accuracy of 96.73 %. Hence, Support Vector Regression is the most robust and precise method for predicting tensile strength.

**Table 7:** Evaluation metrices of Regression algorithms for tensile strength prediction.

Model	R² Value	MAE	MSE	RMSE	MAPE	Accuracy
Linear Regression	0.7438	20.04	568.91	23.85	5.20 %	94.80 %
Random Forest Regression	0.8255	15.41	387.55	19.69	3.84 %	96.16 %
Support Vector Regression	0.8750	12.93	277.51	16.66	3.27 %	96.73 %

**Fig. 12:**
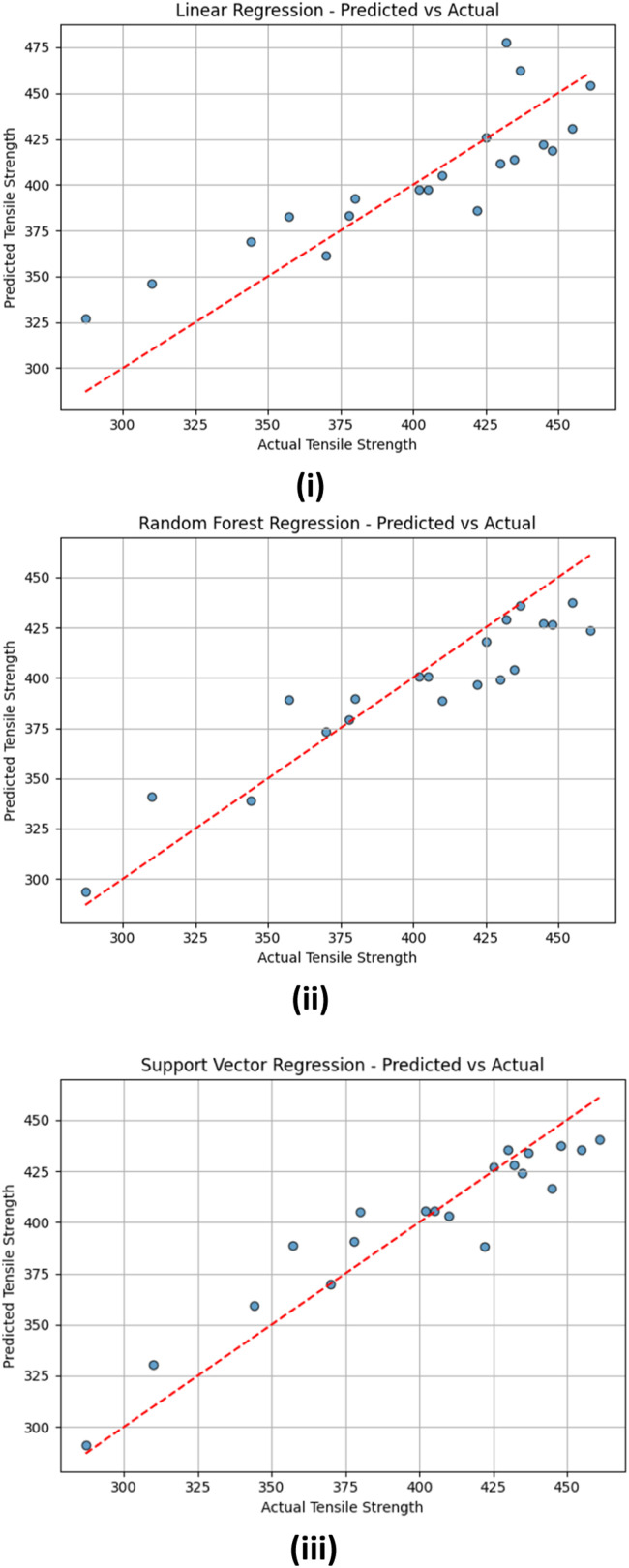
Actual vs Predictred Tensile Strength for i. Linear Regression, ii. Random Forest Regression, iii. Support Vector Regression models.

Fig. 12 depicts a comparison of actual tensile strength measurements and those predicted by three distinct regression models: (i) Linear Regression, (ii) Random Forest Regression, and (iii) Support Vector Regression. In every graph, the red dashed line signifies the perfect scenario where the predicted values align perfectly with the actual results, representing the line of equality. In Fig. 12 (i), the Linear Regression model demonstrates a general correlation between the predicted values and the actual tensile strength measurements. Nevertheless, noticeable deviations from the diagonal line can be seen in lower and higher ranges, indicating that the model tends to underestimate or overestimate in specific areas. This pattern implies that the linear model may not adequately account for the nonlinear aspects present in the dataset.

In Fig. 12 (ii), which showcases the performance of the Random Forest Regression model, the data points are more closely grouped around the ideal diagonal line. This suggests enhanced prediction precision and a superior fit to the data compared to the linear model. The Random Forest model successfully captures intricate relationships due to its ensemble characteristics, although a few outliers are still present, especially in the lower prediction range. Fig. 12 (iii) illustrates that the Support Vector Regression (SVR) model demonstrates a strong correlation between actual and predicted values, with most points near the ideal line. The SVR model can generalize effectively across the data range, providing high prediction accuracy and minimal variation, highlighting its effectiveness in modeling nonlinear relationships.

**Fig. 13:**
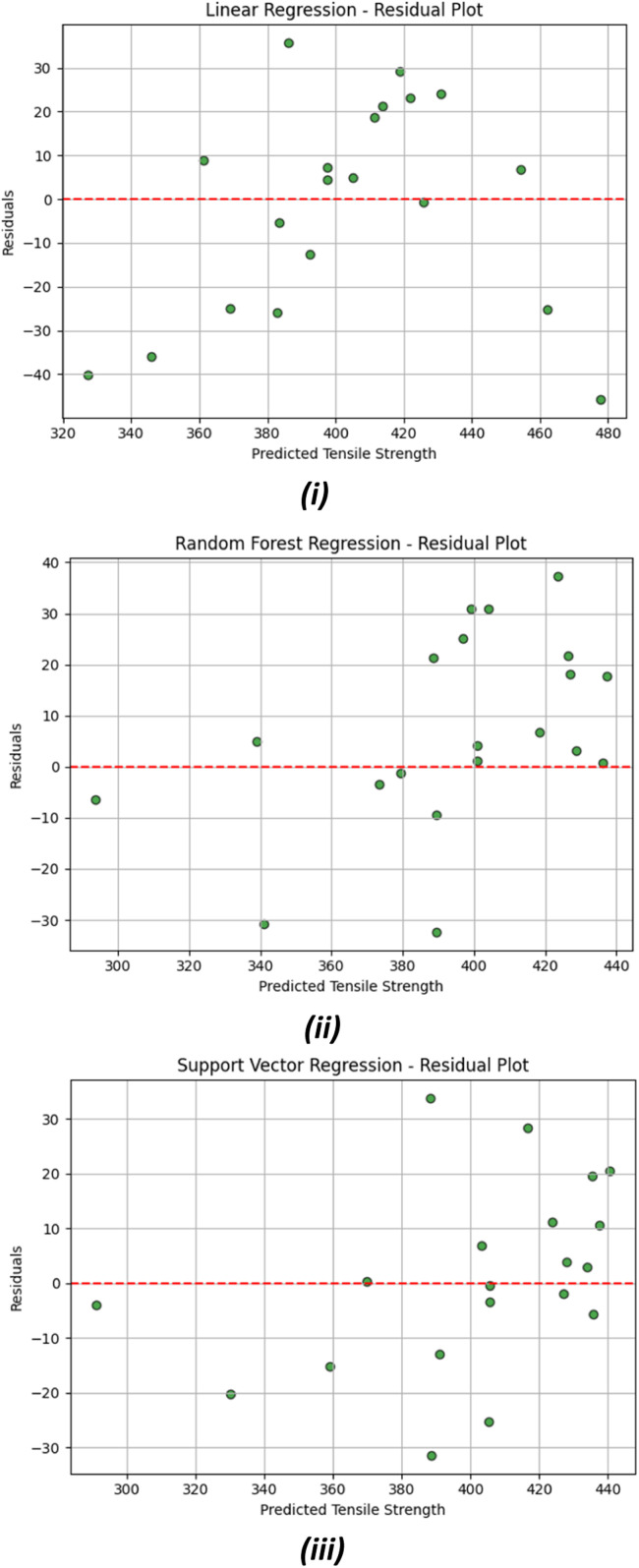
Residuals vs Pridicted Tensile Strength plot for A. Linear Regression, B. Random Forest Regression, C. Support Vector Regression.

Fig. 13 illustrates the residual plots corresponding to the three regression models employed for the prediction of tensile strength: (i) Linear Regression, (ii) Random Forest Regression, and (iii) Support Vector Regression. In these graphical representations, the residuals, the deviations between observed and predicted values, are plotted against the predicted tensile strength values. The red dashed horizontal line denotes the zero-residual baseline, which signifies ideal predictive accuracy.

In the context of linear regression (Fig. 13(i)), the residuals demonstrate a somewhat stochastic distribution both above and below the zero line; however, discernible variation suggests slight heteroscedasticity. This observed pattern may imply that the model inadequately encapsulates the data's intricacies, potentially resulting in underfitting within particular domains.

Regarding the Random Forest Regression model (Fig. 13(ii)), the residuals appear more widely dispersed, particularly at elevated predicted tensile strength values. Although the residuals are predominantly centered around zero, the increasing variability at higher values implies the possibility of overfitting or inconsistent model performance throughout the spectrum of predictions. Nevertheless, the distribution of residuals still corroborates the model's ability to accommodate complex patterns.

The Support Vector Regression model (Fig. 13(iii)) exhibits a more compact and equitable distribution of residuals near the zero line. The reduced variance and more consistent distribution across the prediction range signify a well-generalized model with minimal bias and error.

**Fig. 14:**
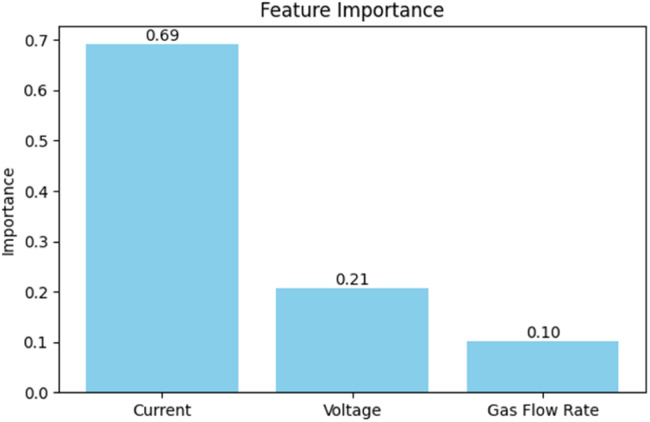
Feature Importance plot.

The feature importance diagram illustrates how much each input variable contributes to predicting output, as plotted in Fig. 14. In the above for support vector regression, Current has the highest importance 69 %, indicating the most important feature for model performance, followed by voltage with 21 %, playing a moderate role, and gas flow rate has the lowest importance only 10 %, suggesting less influence on output.

**How it was calculated:**

1. The Random Forest makes **many decision trees.**
2. Splits the data using Current, Voltage, and Gas Flow Rate to reduce prediction errors in each tree.
3. Measures **how much error is reduced** when using each variable to split the data across all trees.
4. Adds up these reductions for each variable and **divides by the total reduction** to get the **percentage contribution (feature importance)**

**Calculation:**

If Total reduction *T* = 100

Current contributed- 69
Voltage contributed- 21
Gas Flow Rate contributed- 10

These values are then divided by 100 to get the mormalized importance:

Current = 69/100 = 0.69
Voltage = 21/100 = 0.21
Gas Flow Rate = 10/100 = 0.10

**Fig. 15:**
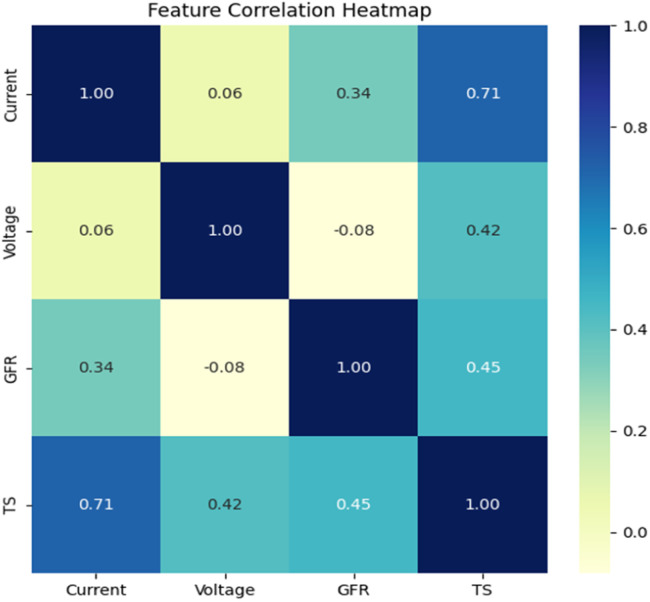
Feature correlation Heatmap.

The feature correlation heatmap illustrates correlation between input variable with output variable, as plotted in Fig. 15. The heatmap shows that current (I) has a strong positive correlation (0.71) with tensile strength, as shown in faint blue, meaning that as current increases, tensile strength also tends to increase. Voltage (V) and gas flow rate (GRF) have a moderate positive correlation (0.42) and (0.45), respectively, as shown in the shade of sky blue. Voltage and GFR still impact tensile strength, but not as much as they do current.

## Conclusion

This study demonstrates that Support Vector Regression (SVR) is a highly effective approach for predicting the tensile strength of EN10028 steel in MIG welding processes. The SVR model achieved a high coefficient of determination (R² = 0.9167) and predictive accuracy of 97.50 %, reflecting its capability to capture the complex, nonlinear relationships between welding parameters namely arc current, arc voltage, and shielding gas flow rate and tensile strength outcomes.

Correlation analysis revealed that welding current exhibits the strongest positive correlation (*r* = 0.71) with tensile strength, while voltage and gas flow rate present moderate correlations, underscoring the critical role of current in influencing weld quality. In contrast, the Linear Regression (LR) model exhibited a lower predictive accuracy of 94.98 %, indicating its limitations in modelling the nonlinearities inherent in MIG welding parameter–tensile strength relationships.

Furthermore, the SVR model demonstrated superior predictive alignment, with predicted tensile strength values closely tracking the experimental results, and residuals distributed compactly around zero, indicating minimal systematic error and robust model stability.

## Limitations

None

## Ethics statements

Our study does not involve human participants and therefore is not considered human subjects research.

## Supplementary material *and/or* additional information [OPTIONAL]

AUDIENCE: This data and method will benefit welding engineers, quality control specialists, and process optimization professionals in fabrication industries such as BHEL, L&T, and Bharat Forge, as well as pipeline and pressure vessel manufacturers. Researchers working on welding process modeling and machine learning applications in manufacturing will also find this methodology useful for predictive maintenance and defect reduction.

## Declaration of competing interest

The authors declare that they have no known competing financial interests or personal relationships that could have appeared to influence the work reported in this paper.

## Data Availability

The data that has been used is confidential.
